# Extraintestinal Pathogenic *Escherichia coli* (ExPEC)-like Infection Associated with Septicemia and Neurological Signs in Suckling Piglets: A Case Report

**DOI:** 10.3390/vetsci13070666

**Published:** 2026-07-09

**Authors:** Annika Gersemann, Gerald Reiner, Sonja von Berg

**Affiliations:** 1 Veterinary Practice Dr. Bernd Kiene & Kollegen GmbH, Am Hövel 12, 49439 Steinfeld-Mühlen, Germany; 2 Department of Veterinary Clinical Sciences, Clinic for Swine, Justus Liebig University Giessen, Frankfurter Strasse 112, 35392 Giessen, Germany; gerald.reiner@vetmed.uni-giessen.de

**Keywords:** swine, *Escherichia coli*, septicemia

## Abstract

**Simple Summary:**

Extraintestinal pathogenic *Escherichia coli* (ExPEC) are uncommon bacteria that can occasionally cause severe systemic disease in pigs. Naturally occurring cases in suckling piglets have rarely been documented. In this case, piglets aged one to three weeks developed neurological signs such as impaired consciousness, abnormal eye movements, unsteady gait, and recumbency. Standard treatment was largely ineffective, whereas early treatment with enrofloxacin improved the condition of some affected animals. Pathological examination revealed inflammation and vascular lesions in the central nervous system, and *E. coli* was isolated from multiple organs. Molecular analyses confirmed the presence of virulence-associated genes typical of ExPEC strains, while other common bacterial causes of neurological disease were excluded. This report documents a septicemic disease with neurological signs in suckling piglets associated with ExPEC-like *E. coli*, and highlights the importance of considering ExPEC-like *E. coli* as a differential diagnosis in piglets with therapy-resistant neurological disorders.

**Abstract:**

Extraintestinal pathogenic *Escherichia coli* (ExPEC) are opportunistic pathogens in pigs and can cause systemic infections with neurological signs. Cases in suckling piglets have rarely been reported. In November 2024, suckling piglets aged 1–3 weeks on a commercial pig farm developed neurological signs, including altered consciousness, vertical nystagmus, ataxic gait, and recumbency. Within two affected batches comprising 1150 and 1200 piglets, 17 and 26 animals, respectively, developed clinical signs (morbidity 1.48% and 2.17%). Initial treatment with amoxicillin and dexamethasone was ineffective. Early administration of intramuscular enrofloxacin combined with dexamethasone was associated with clinical improvement in approximately 50% of affected piglets. Pathological examination revealed atelectatic lung regions, dilated small intestines with yellowish content, and moderate mesenteric vessel congestion. Histologically, the central nervous system of both piglets exhibited hyperemia, edema, disseminated diapedesis hemorrhages, thrombi, and intravascular rod-shaped bacteria. *E. coli* was isolated from the CNS, lungs, pericardium, and joints. Molecular analysis confirmed the presence of virulence-associated genes *fimA*, *fimH*, and *iucD*. Differential diagnostic PCRs for *Streptococcus suis*, *Glaesserella parasuis*, and *Mycoplasma hyorhinis* were negative. This case report documents the first occurrence of an ExPEC-like *E. coli* infection in this herd. The documented clinical, pathological, and molecular findings support the importance of considering ExPEC-like *E. coli* as a differential diagnosis in therapy-resistant neurological diseases in piglets.

## 1. Introduction

Extraintestinal pathogenic *Escherichia coli* (ExPEC) play a key role in human health [1], particularly as causative agents of urinary tract infections [2], invasive sepsis [3], and neonatal meningitis [4], and due to their role as reservoirs of antimicrobial resistance [3,5]. Evidence suggests that human ExPEC isolates may originate from food-producing animals, including pigs, highlighting the relevance of a One Health perspective [6,7].

Reports of ExPEC in pigs have predominantly originated from experimental studies investigating virulence factors and pathogenic mechanisms, or in studies characterizing isolates, regardless of any causality [8,9,10,11,12,13]. In many studies, the pathogenic potential of isolates was inferred from the presence of virulence-associated genes, without direct evidence of pathogenicity in pigs, or without clinical disease being observed in the sampled animals [13,14].

In contrast, only a small number of naturally occurring field cases have been reported in which ExPEC were directly associated with clinical disease in pigs. Nielsen et al. [15] described a fibrinous polyserositis syndrome caused by generalized *E. coli* infection in 1–2-week-old suckling piglets, with a mortality rate of approximately 0.1%. Clinical signs included severe depression, anorexia, rough hair coat, dyspnea, and death within two to five days. Macroscopic lesions were consistent with septicemia, and gelatinous to fibrinous exudate was observed in the pleural, pericardial, and peritoneal cavities. Fibrinous polyarthritis and fibrinous pneumonia were also frequently present. Most of the isolated invasive *E. coli* strains were non-hemolytic, belonged to serogroup O20, and were not further characterized as ExPEC.

Reiner et al. [16] reported a solitary case of hemorrhagic septicemia associated with ExPEC-like virulence factors in an adult pig, and Kizaki et al. [17] described fibrinous meningitis in a small number of suckling piglets. Kong et al. [18] reported an ExPEC isolate from the lung of a pig with pneumonia exhibiting typical ExPEC-associated lesions.

ExPEC can be distinguished from intestinal pathogenic and commensal *E. coli* based on the site of isolation and genetic profiles [19]. Depending on the isolation site, ExPEC isolates in pigs may be categorized analogously to human or avian ExPEC as uropathogenic *E. coli* (UPEC), avian pathogenic *E. coli* (APEC), neonatal meningitis *E. coli* (NMEC), or sepsis-associated *E. coli* (SEPEC). The ability of ExPEC to cause disease in pigs is thought to depend on a combination of extraintestinal virulence factors and complex host–microbe interactions, rather than a single straightforward mechanism [20].

As a species, *E. coli* comprises commensal, intestinal-pathogenic, and extraintestinal-pathogenic lineages distributed across distinct phylogenetic groups (A, B1, B2, D, and others) that differ markedly in genome content and host association [1,19,20]. ExPEC do not form a single clonal group but are defined operationally by the carriage of extraintestinal virulence-associated genes, which are frequently located on mobile genetic elements and pathogenicity islands and can be exchanged between lineages [19,20]. Whole-genome sequencing (WGS) has refined this picture by resolving phylogroup, sequence type, serotype, and virulence- and resistance-gene content at single-nucleotide resolution, and has been applied specifically to porcine ExPEC populations [8,12,18]. These data indicate that swine-associated ExPEC are genetically heterogeneous and partly overlap with isolates from other hosts, which is relevant to host adaptation and to the assessment of zoonotic potential [8].

Coliform bacteria, particularly *E. coli*, including potential ExPEC strains, are frequently associated with mastitis in sows and have repeatedly been detected in mammary secretions of affected animals [21,22].

In contrast, the present case represents a primary ExPEC infection with septicemic dissemination in neonatal piglets and therefore differs fundamentally from maternal inflammatory syndromes with secondary bacterial transmission.

## 2. Case Presentation

### 2.1. Case History

The affected farm maintains 700 sows under a closed system, operating on a three-week batch production schedule with a four-week lactation period. Farrowing units follow an all-in/all-out system with a four-day service period for cleaning and disinfection prior to new occupancy. The genetics used are Danbred × PIC 408. Semen is sourced from a certified boar stud, and replacement gilts are purchased at 30 kg.

Piglets receive an iron supplement, PRRSV vaccination (Unistrain, Hipra, Düsseldorf, Germany), and a vaccine against Shiga toxin (Ecoporc Shiga, Ceva, Düsseldorf, Germany) on day 4 of life. Ear tagging is also performed at this age. Other routine interventions, including teeth grinding and castration, are not performed. On days 14 and 26, piglets are vaccinated against PCV2 and *Mycoplasma hyopneumoniae* using a combination vaccine (Porcilis PCV Mhyo, MSD, Unterschleissheim, Germany), and receive farm-specific vaccination against *Glaesserella parasuis*, *Mycoplasma hyorhinis*, and *Streptococcus suis* (San Group Biotech, Höltinghausen, Germany). Needle change is performed per litter. Feeding in all units is with commercial complete feed.

### 2.2. Clinical Findings

The first clinical signs were observed in November 2024. In two farrowing groups comprising 1200 and 1150 piglets, 26 and 17 animals were affected, respectively. These piglets were between 1 and 3 weeks of age, well-nourished, and of normal general condition. No association was observed between disease occurrence and sow parity. Typically, only one to three piglets per litter were affected. Clinical signs varied considerably. Some piglets showed neurological symptoms and were found in lateral recumbency with paddling movements and, in some cases, vertical nystagmus. Others exhibited ataxia, generalized tremors, and disorientation within the farrowing pen, showing absent or markedly reduced menace responses. Other piglets showed exclusively orthopedic signs. These were predominantly found in sternal recumbency and, when prompted, some were barely able to walk or stand, displaying a hunched back. These animals immediately resumed a recumbent position. Rectal temperatures ranged from 39.8 °C to 40.2 °C. Fecal and urinary outputs were unremarkable.

Morbidity varied between 1.48% and 2.17% per group. Based on the clinical presentation and farm history of *S. suis*-associated meningitis and arthritis, an initial presumptive diagnosis of *S. suis* infection was made. Treatment with amoxicillin in combination with dexamethasone showed no clinical effect. The occurrence of this syndrome and its poor response to initial therapy had not previously been observed in the herd.

Differential diagnoses included *G. parasuis*, *M. hyorhinis* infections and edema disease. Considering the intrinsic resistance of *M. hyorhinis* to beta-lactams, therapy was adjusted. Early administration of intramuscular enrofloxacin combined with dexamethasone was associated with clinical improvement in approximately 50% of affected piglets. Concurrently, untreated piglets displaying typical clinical signs were euthanized and directly submitted to an external laboratory for necropsy and further examination (San Group Biotech, Höltinghausen, Germany).

### 2.3. Gross Pathological Findings

Two affected piglets underwent full necropsy, and targeted organ samples were collected from three additional piglets from two farrowing groups.

Gross pathological examination revealed small atelectatic regions in the middle lung lobes of both animals. One piglet exhibited gastric mucosal hyperkeratosis in the pars esophagea. The small intestine contained thin, yellowish contents with moderately congested mesenteric vessels. The large intestine contained moderately desiccated greenish content. No additional gross lesions were detected.

Samples were collected from the CNS, pericardium, pleura, humeroulnar joint and both tarsal joints for bacteriological and molecular analysis.

### 2.4. Histopathological Findings

Histopathological examination of the CNS in both necropsied piglets revealed the following:

- Piglet 1: Moderately acute, diffuse hyperemia of the cerebral cortex with disseminated mild diapedesis hemorrhages in the meninges, white and grey matter. Occasional hyaline thrombi, endothelial cell necrosis, and sparse intravascular rod-shaped bacteria were observed.
- Piglet 2: Moderately acute diffuse hyperemia and edema with numerous intravascular rod-shaped bacteria throughout the CNS.

### 2.5. Microbiological and Molecular Findings

Laboratory findings showed *E. coli* in the CNS, pericardium, pleura, and lung of both necropsied animals. PCR analysis confirmed the presence of virulence-associated genes *fimA*, *fimH*, and *iucD*. No additional bacterial species were reported.

Targeted organ sampling also yielded *E. coli* from CNS, tarsal joints and the left humeroulnar joint, all harbouring the same virulence genes (*fimA*, *fimH*, *iucD*).

In both examinations, *E. coli* was the only microorganism isolated from the examined organs. The CNS was additionally tested by PCR for pathogens relevant to the differential diagnosis (*S. suis*, *G. parasuis*, *M. hyorhinis* and EDEC, i.e., edema disease *E. coli* detected via the *stx2e* gene). These investigations revealed negative results.

Data from the susceptibility testing are presented in Table 1.

### 2.6. Diagnosis

Based on bacterial isolation from multiple organs, detection of *fimA*, *fimH*, and *iucD*, and histopathological findings, a diagnosis of infection with *E. coli* harbouring ExPEC-associated virulence genes was established.

### 2.7. Therapy and Prophylaxis

Due to increasing mortality and morbidity, affected piglets in the first three weeks of life were treated with enrofloxacin according to susceptibility testing.

Based on the diagnostic results, an autogenous vaccine was updated to include the identified *E. coli* (San Group Biotech, Höltinghausen, Germany). The vaccine was administered to sows at 6 and 3 weeks antepartum. In the subsequent farrowing group, antibiotic therapy was no longer required. Morbidity decreased to 0.16% and mortality to 0.08%. Clinical stability was maintained in subsequent batches. In addition to the management measures already implemented on this farm (abandonment of castration and teeth grinding, needle changes between each litter), split-suckling management was implemented to ensure adequate colostrum intake for each piglet. Because the updated autogenous vaccine was introduced together with these management changes and against the background of a sporadic, self-limiting problem, the subsequent decline in morbidity and mortality cannot be attributed to vaccination alone.

## 3. Discussion

Septicemia associated with ExPEC in pigs appears to be exceedingly rare with only a few documented field cases [15,16,17,18]. The primary route of infection is via oral uptake, though infections via the respiratory tract or umbilicus are possible [23]. Risk factors include poor hygiene, inadequate colostrum intake, and open wounds from routine procedures such as incorrect teeth grinding, tail docking, or castration [23]. General hygiene standards are high and the transmission of pathogens via open wounds or contaminated needles is already actively minimized on this farm. Inadequate colostrum intake may therefore have represented a potential risk factor in this outbreak.

The isolation of *E. coli* from the CNS, pericardium, pleura, lungs, and joints, together with the presence of the ExPEC-associated virulence factor *iucD* (aerobactin), supports a septicemic infection. The observed predominantly vascular and septicemic CNS lesions align with the findings reported by Kizaki et al. [17], while the involvement of the lungs corresponds to the observations of Kong et al. [18]. The clinical picture most closely resembles that described by Nielsen et al. [15], who also reported mortality, depression, anorexia, rough hair coat, dyspnea, septicemia, fibrinous pleuritis, pleuropneumonia, peritonitis, and polyarthritis. Unlike the case reported by Reiner et al. [16], no hemorrhages were observed in the present case.

Clinical signs may occasionally resemble crush injuries; however, the age distribution and location of affected piglets made this diagnosis unlikely. Differential diagnoses include *Streptococcus suis* (meningitis, arthritis), *Glaesserella parasuis* (polyarthritis, meningitis), and *Mycoplasma hyorhinis* (polyserositis, arthritis, occasional meningitis) [24,25,26,27]. In this case, cultures and PCRs for these pathogens were negative.

Shiga toxin-producing *E. coli* (STEC) were not investigated; however, STEC-associated disease typically occurs after the fourth week of life, and tissue lesions are toxin-mediated rather than due to bacterial colonization. Moreover, the piglets had been vaccinated against Shiga toxin (Ecoporc Shiga) on day 4 of life, and the differential PCR for edema disease *E. coli* (*stx2e*) performed on the CNS was negative, further reducing the likelihood of a Shiga toxin-associated process. Immunosuppressive viruses such as PRRSV and PCV2, which may predispose piglets to secondary infections, were regularly monitored and consistently negative. Pseudorabies (Aujeszky’s disease) virus was not tested in brain tissue, as the domestic pig population in Germany holds official Aujeszky’s disease-free status; in addition, the predominantly vascular, bacteria-associated CNS lesions, together with the isolation of *E. coli* from the central nervous system, are not consistent with a primary herpesviral encephalitis.

Therapeutic options for septicemia are limited. Prompt antibiotic treatment is essential, ideally guided by an antibiogram due to frequent multidrug resistance [7]. Therapy is most effective before clinical signs develop; in this outbreak, only approximately 50% of affected piglets responded. Consequently, metaphylactic treatment of clinically healthy littermates may be warranted. Because fluoroquinolones are classified as critically important antimicrobials, their use in this outbreak was reserved for piglets with life-threatening systemic disease after failure of first-line beta-lactam therapy and was guided by susceptibility testing; such restraint is essential to limit the selection of resistance. Septicemic ExPEC infections are not typically considered target diseases for control using autogenous bacterins. Recurrent herd problems have not been reported, and the influence of a single transient illness in one group on the overall disease occurrence is considered unlikely as the clinical presentation extended across multiple farrowing groups. However, the extent to which the vaccination contributed to the resolution of the issue in the present case could not be established. Currently, to our knowledge, there is no peer-reviewed, published vaccine study targeting extraintestinal *E. coli* in pigs. The extremely rare and case-specific presentation of ExPEC disease in swine supports the notion that individual, and hitherto unclear, predisposing conditions may be crucial in permitting such events to occur. It remains unclear why only a small number of animals were affected, both in the present case and in previously reported outbreaks [15,17]. Moreover, no evidence of a potential source of introduction could be identified.

From a One Health perspective, ExPEC are relevant beyond the individual herd: food-producing animals, including pigs, have been proposed as reservoirs of ExPEC lineages and of ExPEC-associated virulence and resistance genes that also occur in human extraintestinal infections [1,6,7]. While the direction and magnitude of any pig-to-human transmission remain debated and were not investigated here, the carriage of mobile resistance determinants by invasive porcine *E. coli* is of interest to both animal and public health and reinforces the need for prudent, diagnostically guided antimicrobial use. At the same time, these considerations should not be over-extended: a single herd-level case does not establish a broader epidemiological or zoonotic trend.

This report has several limitations. Isolate characterization was restricted to three virulence-associated genes (*fimA*, *fimH*, *iucD*); serotyping, phylogrouping, multilocus sequence typing, whole-genome sequencing, an extended ExPEC virulence-gene panel, and plasmid and resistome analyses were not performed, so the organism is most accurately described as *E. coli*-harbouring ExPEC-associated virulence genes rather than as a comprehensively characterized ExPEC strain. Detection of these genes by PCR documents their presence but not their expression or functional contribution to disease. Full necropsy was available for only two piglets, with targeted organ sampling from three additional animals, and the identity of the isolates from different organs was not confirmed by molecular typing; the inference of septicemic dissemination therefore rests on repeated isolation of *E. coli* with the same gene profile rather than on demonstrated clonality. The CNS lesions were predominantly vascular and septicemic, and a primary suppurative meningoencephalitis was not established. Antimicrobial susceptibility results for agents lacking *E. coli*-specific clinical breakpoints should be regarded as descriptive only (Table 1). All conclusions should be interpreted in proportion to these constraints, and whole-genome sequencing would be required to provide definitive characterization of the isolate’s pathogenic potential. As the case was investigated by an external diagnostic laboratory as part of the clinical work-up, additional or retrospective testing beyond that reported here was not feasible.

This case underscores the extreme rarity of ExPEC infections in pigs and highlights the importance of rigorous differential diagnostics, appropriate management practices, and rapid intervention when septicemic piglets are encountered.

## 4. Conclusions

*E. coli*-harbouring ExPEC-associated virulence genes can, in rare instances, be associated with systemic disease in suckling piglets, thereby expanding the range of differential diagnoses to be considered. Individual risk factors, such as insufficient colostrum intake, hygiene deficits, or minor injuries, may predispose affected piglets. Nevertheless, these infections remain extremely sporadic, and outbreaks are not expected to occur frequently.

Awareness of ExPEC-like *E. coli* as a potential, though uncommon, pathogen may aid in the diagnosis and management of atypical septicemic cases in piglets.

## Figures and Tables

**Table 1:** Minimum inhibitory concentrations (MICs) and antimicrobial susceptibility of the ExPEC isolate obtained from suckling piglets with septicemia and neurological signs.

Antimicrobial Class	Antimicrobial Agent	MIC (µg/mL)	Interpretation
β-Lactams	Amoxicillin/ Clavulanic acid	8/4	Susceptible
	Ampicillin	>16	Resistant
	Ceftiofur	0.5	Susceptible
	Penicillin	>2	Resistant
Fluoroquinolones	Enrofloxacin	0.0625	Susceptible
Aminoglycosides	Gentamicin	0.5	Susceptible
Macrolides/ Pleuromutilins	Erythromycin	>4	Resistant
	Tiamulin	>16	Resistant
	Tilmicosin	>16	Resistant
	Tulathromycin	4	Susceptible
	Gamithromycin	2	Susceptible
Amphenicols	Florfenicol	4	Intermediate
Polymyxins	Colistin	≤0.5	Intermediate
Sulfonamides/ Diaminopyrimidines	Trimethoprim/ sulfamethoxazole	>2/38	Resistant
Tetracyclines	Tetracycline	>8	Resistant

Interpretation note: The susceptibility categories should be regarded as descriptive only and not as clinically predictive.

## Data Availability

The original contributions presented in this study are included in the article. Further inquiries can be directed to the corresponding author.

## Abbreviations

The following abbreviations are used in this manuscript:

APEC	Avian pathogenic *Escherichia coli*
CNS	Central nervous system
ExPEC	Extraintestinal pathogenic *Escherichia coli*
*E. coli*	*Escherichia coli*
*G. parasuis*	*Glaesserella parasuis*
*M. hyopneumoniae*	*Mycoplasma hyopneumoniae*
*M. hyorhinis*	*Mycoplasma hyorhinis*
NMEC	Neonatal meningitis *Escherichia coli*
PCV2	Porcine circovirus type 2
PCR	Polymerase chain reaction
PRRSV	Porcine reproductive and respiratory syndrome virus
SEPEC	Sepsis-associated *Escherichia coli*
*S. suis*	*Streptococcus suis*
STEC	Shiga toxin-producing *Escherichia coli*
UPEC	Uropathogenic *Escherichia coli*
